# Metabolomic Approach for Discrimination of Cultivation Age and Ripening Stage in Ginseng Berry Using Gas Chromatography-Mass Spectrometry

**DOI:** 10.3390/molecules24213837

**Published:** 2019-10-24

**Authors:** Seong-Eun Park, Seung-Ho Seo, Eun-Ju Kim, Dae-Hun Park, Kyung-Mok Park, Seung-Sik Cho, Hong-Seok Son

**Affiliations:** 1 School of Korean Medicine, Dongshin University, Naju 58245, Korea; seong9525@naver.com (S.-E.P.); blue784300@naver.com (S.-H.S.); dhj1221@dsu.ac.kr (D.-H.P.); parkkm@dsu.ac.kr (K.-M.P.); 2 Department of Pharmacy, College of Pharmacy, Mokpo National University, Mokpo 58554, Korea; sscho@mokpo.ac.kr

**Keywords:** ginseng berry, cultivation age, ripening stage, metabolomics, random forest

## Abstract

The purpose of this study was to analyze metabolic differences of ginseng berries according to cultivation age and ripening stage using gas chromatography-mass spectrometry (GC-MS)-based metabolomics method. Ginseng berries were harvested every week during five different ripening stages of three-year-old and four-year-old ginseng. Using identified metabolites, a random forest machine learning approach was applied to obtain predictive models for the classification of cultivation age or ripening stage. Principal component analysis (PCA) score plot showed a clear separation by ripening stage, indicating that continuous metabolic changes occurred until the fifth ripening stage. Three-year-old ginseng berries had higher levels of valine, glutamic acid, and tryptophan, but lower levels of lactic acid and galactose than four-year-old ginseng berries at fully ripened stage. Metabolic pathways affected by different cultivation age were involved in amino acid metabolism pathways. A random forest machine learning approach extracted some important metabolites for predicting cultivation age or ripening stage with low error rate. This study demonstrates that different cultivation ages or ripening stages of ginseng berry can be successfully discriminated using a GC-MS-based metabolomic approach together with random forest analysis.

## 1. Introduction

Ginseng root (*Panax ginseng* Meyer) has long been widely used in Asia as a traditional medicinal herb. Ginsenosides are the principal effective components of ginseng roots. They show medicinal effects for various diseases, such as hypertension [1], diabetes [2], asthma [3], and cancer [4]. Although most ginseng studies have focused on ginsenosides of ginseng roots, ginsenosides are also distributed in other parts of ginseng plant, including leaf and berry. It has been reported that contents of ginsenosides in ginseng berries (GBs) are higher than or similar to those in ginseng roots [5]. In addition, several studies have shown that the profile of ginsenosides in GBs differs from that in ginseng roots [6,7]. For example, GBs are known to have ginsenosides F1, F2, and F3 that are not present in ginseng roots [8,9]. According to some studies [10,11,12], GBs have more potent pharmacological properties than ginseng roots.

Medicinal properties of ginseng are primarily due to the presence of ginsenosides. Therefore, previous GB studies have mainly focused on the identification and quantification of ginsenosides. In recent years, as the efficacy of GB has become well-known, the use of GB as food such as juice has increased. Thus, information regarding the primary metabolites in GB, such as amino acids and organic acids, and sugars related to its organoleptic properties and functional components are important. Recently, metabolomics studies have successfully revealed that metabolic profiles of ginseng vary according to different ripening stage [13], geographical origin, [14] tissue [15], and cultivar [16] from a holistic perspective.

Several studies have reported that pharmacological compositions and effects of ginseng differ depending on the age of cultivation [17,18]. Likewise, contents of ginsenosides in GB can be different depending on the cultivation age. Song et al. [19] have reported that the contents of ginsenosides except Rg1 tend to be higher in four-year-old GB than those in three-year-old GB. As the cultivation age of ginseng can hardly be determined by physical appearance, a reliable method to discriminate the cultivation age of ginseng is required. One study has shown that different cultivation age of ginseng can be successfully discriminated by a metabolomic approach [20]. However, studies on GB according to cultivation age are insufficient. The ripening stage of GB is considered another important factor in determining the quality and efficacy of fruit as in all plants. Lee et al. [21] have reported that most ginsenosides are relatively abundant in GB during preharvest stages.

Recently, metabolomics studies have revealed that the metabolic profile of GB varies according to different ripening stages using liquid chromatography (LC), gas chromatography (GC)-mass spectrometry (MS) [21] and proton nuclear magnetic resonance (^1^H NMR) [13,22]. However, no single analytical platform can observe all metabolites in a sample. Thus, multiple analytical platforms should be used to extend the range of GB metabolome. GC-MS in combination with derivatization is more suitable for analyzing small polar metabolites covering more primary metabolism than other methods [23]. To propose a clear age discrimination method for GB, studies using samples of various ripening stages should be conducted.

Thus, the objective of the present study was to investigate metabolite changes of GB during five different ripening stages of three-year-old and four-year-old ginseng. Moreover, a random forest machine learning approach was applied to obtain a predictive model for cultivation age and to identify important metabolites for separation. 

## 2. Results and Discussion

### 2.1. Metabolic Profiling of GB with Different Cultivation Ages and Ripening Stages

**Figure 1:**
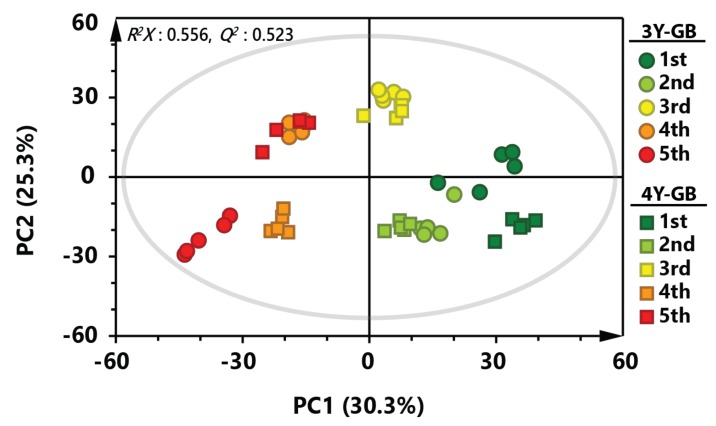
Principal component analysis (PCA) score plot derived from gas chromatography-mass spectrometry (GC-MS) data of GB at different ripening stages. Circular symbols and rectangular symbols represent three-year-old GB and four-year-old GB, respectively. The color of each symbol is as follows: green color, first harvested sample; light green, second harvested sample; yellow color, third-harvested sample; orange color, fourth-harvested sample; red color, fifth-harvested sample.

PCA was applied to investigate overall metabolic profiles between GB samples with different cultivation ages and ripening stages (Figure 1). PCA score plot of GB samples showed a separation pattern by the ripening stage, indicating that metabolite profiles of early harvested GB samples were largely different from those of late harvested GB samples (*R^2^X* = 0.556, *Q^2^* = 0.523). Metabolic profiles of three-year-old GB samples showed similar patterns to those of four-year-old GB samples until the third ripening stage. However, fourth- and fifth-harvested GB samples were clearly separated by cultivation age. Interestingly, fourth-harvested three-year-old GB samples and fifth-harvested four-year-old GB samples were clustered in the similar position on the PCA score plot, suggesting that metabolites of GBs might be different not only by ripening stage, but also by cultivation age.

### 2.2. Metabolic Changes of GB by Ripening Stage

**Figure 2:**
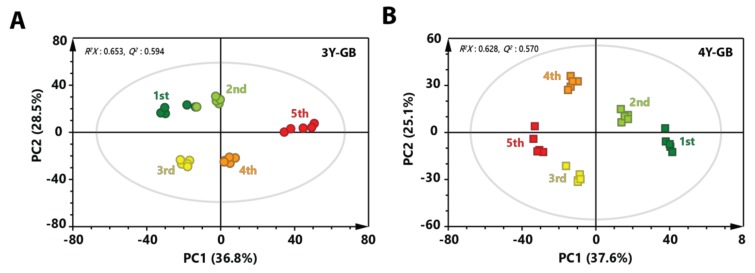
PCA score plot derived from GC-MS data of (**A**) three-year-old GB and (**B**) four-year-old GB with different ripening stages. Circular symbols and rectangular symbols represent three-year-old GB and four-year-old GB, respectively. The color of each symbol is as follows: green color, first harvested sample; light green, second-harvested sample; yellow color, third-harvested sample; orange color, fourth-harvested sample; red color, fifth-harvested sample.

To better investigate metabolic changes at different maturity stage of GB, PCA models were applied using features (metabolites) from GB samples having the same cultivation age with different ripening stages (Figure 2). A clear separation was shown at each ripening stage in the score plot, indicating that continuous metabolic changes occurred until the fifth ripening stage. Generally, GBs were harvested at the end of July in Korea. In this study, fourth and fifth-harvested GB samples were at fully ripe stage. These results indicate that metabolites in GB might change depending on ripening stage.

A total of 20 metabolites were identified in GB samples based on fragmentation patterns of NIST library, retention index, and an in-house library of our lab made by standard chemicals. Metabolites identified in this study included some amino acids, organic acids, and sugars, consistent with recently reported results of metabolic changes of GB during maturation stages [16,21,22]. Table 1 shows changes of these identified metabolites during ripening of GB.

Fruit ripening is generally characterized by increased levels of sugars coupled with decreased levels of organic acids. As expected, as GB ripened, levels of xylose, fructose, glucose, and galactose increased. On the other hand, levels of lactic acid, glycolic acid, malonic acid, succinic acid, and glyceric acid showed a tendency to decrease. However, levels of malic acid increased in both three-year-old and four-year-old GBs, unlike the general expectation.

Most amino acids detected in this study showed a decreasing pattern in late harvested GB samples. However, except for glutamic acid, no amino acid showed significant difference according to ripening stage in three-year-old or four-year-old GB. Interestingly, as GB ripened, levels of tryptophan and glycerol increased in three-year-old GB, but decreased in four-year-old GB. According to Lee et al. [21], most amino acids and organic acids were higher in the early maturation stage of GB than those in the late maturation stage. Yang et al. [22] have reported that GB samples harvested at different ripening stages could be clearly distinguished by their different levels of amino acids profiles.

### 2.3. Metabolic Differences Between Three-Year-Old GB and Four-Year-Old GB

**Figure 3:**
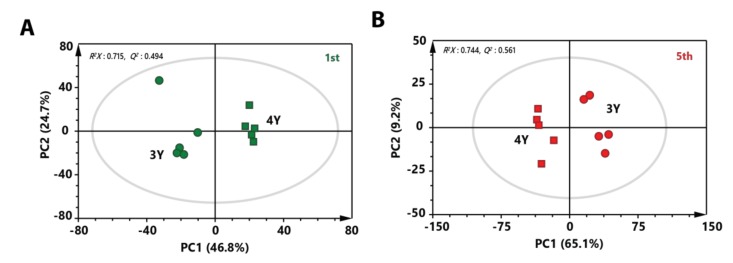
PCA score plot derived from GC-MS data between three-year-old GB and four-year-old GB at (**A**) the first harvest time and (**B**) the fifth harvest time. Circular symbols and rectangular symbols represent three-year-old GB and four-year-old GB, respectively. The color of each symbol is as follows: green color, first-harvested sample; red color, fifth-harvested sample.

To investigate metabolic differences between three-year-old GB and four-year-old GB harvested at the same stage, PCA score plots were made. A clear separation between three-year-old GB and four-year-old GB was observed in both score plots of immature and mature stages (Figure 3A,B). These results indicate that metabolite profiles of three-year-old GB were different from those of four-year-old GB. To identify metabolites that affected separation between three-year-old GB and four-year-old GB in fully ripened stage, variable importance in projection (VIP) score was determined by partial least squares-discriminant analysis (PLS-DA). Based on a VIP score higher than 1.0 with lower *p* value than 0.05 in two-tailed Student’s *t*-test, a total of 16 metabolites affecting the differentiation were identified.

**Figure 4:**
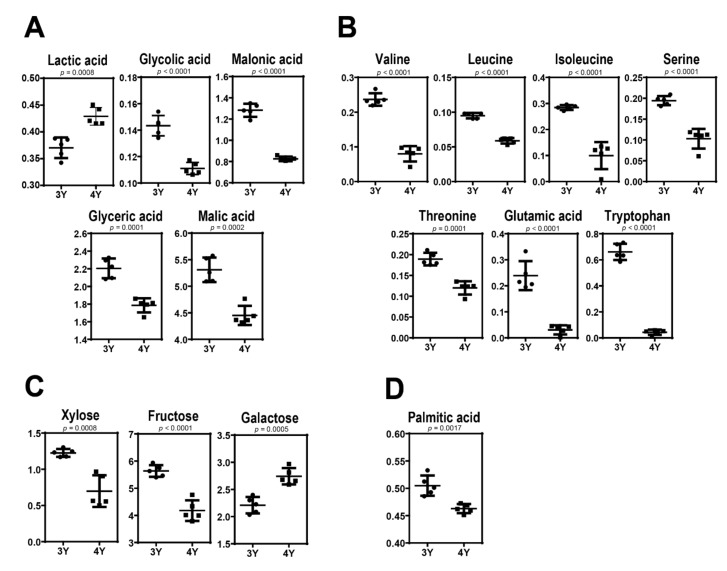
Scatter dot plots in levels of (**A**) organic acids, (**B**) amino acids, (**C**) sugars, and (**D**) fatty acid between three-year-old GB and four-year-old GB at the fifth harvest time.

Figure 4 shows quantitative differences of these identified metabolites in GB samples harvested at the fully ripened stage. Three-year-old GBs were found to have higher levels of glycolic acid, malonic acid, malic acid, valine, leucine, isoleucine, serine, threonine, glutamic acid, tryptophan, xylose, fructose, glyceric acid, and palmitic acid but lower levels of lactic acid and glucose compared to four-year-old GBs. These results indicate that GB with different characteristics can be obtained according to cultivation age.

**Figure 5:**
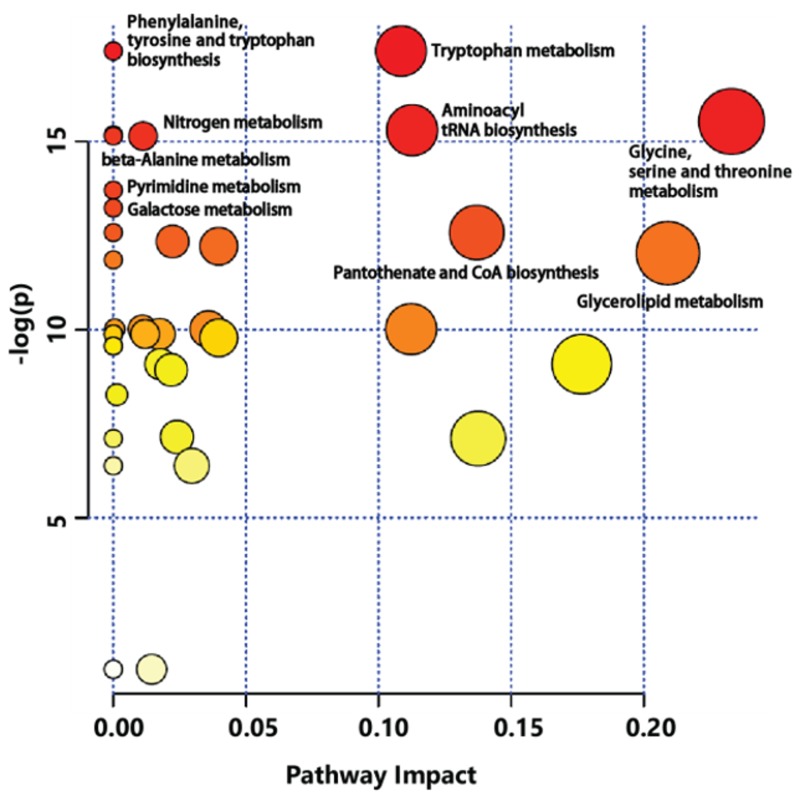
Metabolic pathway analysis (MetPA) displaying metabolic pathways arranged by scores from pathway enrichment (y axis) and from topology analysis (x axis) using MetaboAnalyst to identify the most relevant pathways between three-year-old GB and four-year-old GB. All matched pathways are displayed as circles. Color and size of circles indicate significant changes in metabolites based on *p* value and pathway impact score in metabolic pathways, respectively.

Metabolic pathway analysis was conducted to identify relevant metabolic pathways of GB affected by cultivation age using the MetPA tool of MetaboAnalyst (Figure 5). This analysis shows pathways based on *p* values by enrichment analysis. It also shows impact values by topology analysis. Metabolic pathways affected by different cultivation ages of GB were involved in amino acid metabolism pathway. Significant differences were observed for specific metabolites connected to tryptophan metabolism, phenylalanine-tyrosine and tryptophan biosynthesis, glycine-serine and threonine metabolism, aminoacyl-tRNA biosynthesis, nitrogen metabolism, beta-alanine metabolism, and so on. This finding was supported by different levels of amino acids between three-year-old and four-year-old GBs.

GB can be harvested several times from 3–6 years of growth, because ginseng flowers and fruits appear from the third year [7]. Generally, GBs are collected when plants are 3–4 years old for the purpose of seed harvesting [24]. Seed harvesting of ginseng is conducted only once during the growth period of ginseng. This is because harvesting seeds for more than two times will greatly reduce the yield and quality of ginseng. Although seeds harvested from three-year-old ginseng are good for seed germination; they are less likely to be harvested because these seeds are small. On the other hand, although seeds over five years old are able to germinate, the success rate of germination of seeds is reduced because of the thick surface of GB. For that reason, it would be better to use four-year-old seeds to get GB for the purpose of seed harvesting. However, in recent years, GB is not only used for harvesting seed, but also consumed in tea or juice as its ingredients and efficacy are becoming well-known. From this point of view, since most metabolites, such as organic acids, amino acids, and sugars, in the three-year-old GB are higher at the fully ripened stage than those in the four-year-old GB, three-year-old GB can be appropriate for fruit consumption.

### 2.4. Classification of GB by Random Forest Machine Learning Algorithm

**Figure 6:**
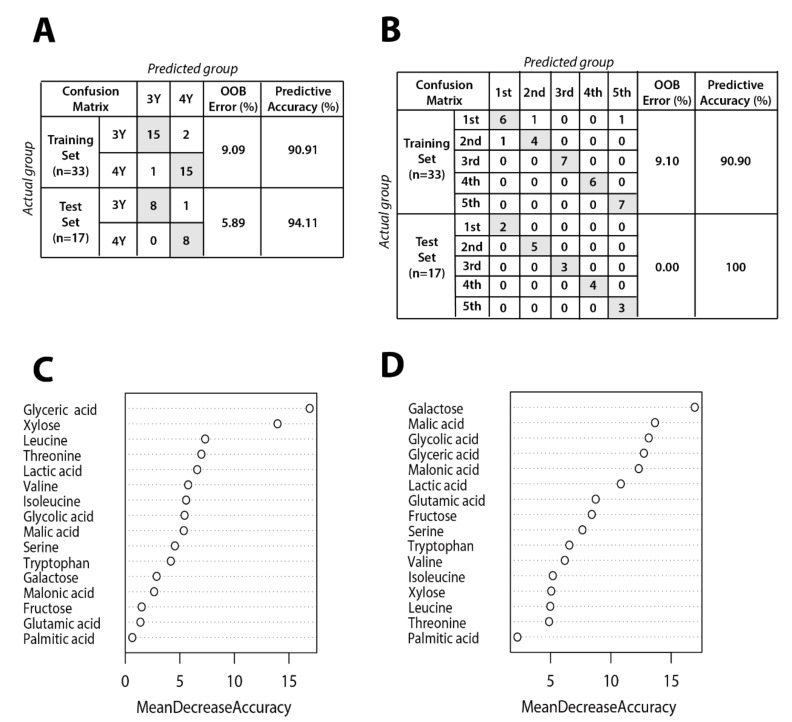
Random forest model performance comparison between actual group and predicted group for (**A**) cultivation age and (**B**) ripening stage. Panels (**C**) and (**D**) show mean decrease accuracy measuring the performance of the model without each metabolite for cultivation age and ripening stage, respectively. The y-axis of variable importance plots represents metabolites in order of importance for group classification.

Figure 6 shows results of random forest classification according to GB growth and ripening stage. The dataset was randomly divided into training (66%, *n* = 33) and testing (34%, *n* = 17) data. The random forest model was generated using a training set including 16 metabolites affecting the differentiation between three-year-old GB and four-year-old GB. Metabolites data for the prediction models of ripening stage and cultivation age are shown in Table S1. Out-of-bag (OOB) error rates of the prediction model for cultivation age and ripening stage were only 9.09% and 9.10%, respectively. To test the reproducibility of the prediction model trained on data from 33 GB samples, an independent data set from 17 GB samples was tested. The OOB error rates of the prediction model for cultivation age was 5.99%, lower than for the model by training set. Interestingly, the OOB error rate of the prediction model for ripening stage was 0%.

The low OOB error rate for cultivation age model indicates that three and four-year-old GB can be distinguished regardless of ripening stage. The importance of each metabolite in the random forest model was evaluated by mean decrease accuracy method. Metabolites that are important for separation according to cultivation age were glyceric acid, xylose, leucine, threonine, and so on. Metabolites contributing to separation according to ripening stage were identified as galactose, malic acid, glycolic acid, glyceric acid, and so on. These metabolites could potentially serve as biomarkers to distinguish between GB samples according to ripening stage and cultivation age.

Although xylose and galactose are both sugar, xylose was more important in sample differentiation by the age while galactose was more important in sample differentiation by the ripening stage. Although many studies have revealed metabolic changes during fruit ripening, different results have been reported for different fruit species. Osorio et al. [25] have reported that amino acid profiles during development and ripening of pepper and tomato were different presumable due to distinct metabolic regulation programs. According to Robert et al. [26], the changes of monosaccharide, including xylose and galactose, were different in the nine fruits after ripening. Furthermore, few studies have examined the differences in metabolite levels by the cultivation age. As plant hormones play critical roles in plant growth and development and fruit ripening [27], metabolomics studies involving plant hormones are necessary to understand metabolic changes during development and ripening.

Recently, machine learning methods using output data from metabolite analysis have been used to accurately separate ginseng samples. Kim et al. [20] reported that different cultivation ages of ginseng can be successfully separated by the metabolomic approach, together with classification methods of random forest, prediction analysis of microarray, and PLS-DA. According to Song et al. [14], the prediction of geographical origins of Korean ginseng can be successfully performed at 99.7% probability by the orthogonal projections to latent structures-discriminant analysis (OPLS-DA) model. A machine learning method should consider various factors for a single data set because metabolites of GB can vary depending on ripening stage, growth location, cultivation age, and other factors. The algorithm generated in this study can be applied to the determination of actual cultivation age because samples from different ripening stage are used. Further investigations of various GB samples should be performed to build a more robust model. Moreover, this method could contribute to the identification of potential biomarkers for each age and ripening stage.

## 3. Materials and Methods

### 3.1. Plant Materials and Preprocessing

GBs were harvested from a local farm (Healthy Sam-Farm, Iksan, Korea) every week from July 1 to July 30, 2017. To ensure biological replications, five different GBs were randomly collected from different trees of three-year-old and four-year-old ginseng, respectively, within the same ginseng field. Each GB was dried, and then extracted with 70% ethanol at room temperature for 72 h. After removing residual ethanol, GB samples were freeze-dried, and stored at −80 ℃ for analysis.

### 3.2. Sample Derivatization

Sample derivatization procedures for GC-MS analysis were similar to those described in a tutorial metabolomics study [28] and our previous study [29]. Briefly, 100 μL of O-methoxyamine hydrochloride in pyridine (15 mg/mL) was added to each lyophilized sample. After vortex mixing for 10 min, the mixture was incubated at 25 ℃ for 16 h. Subsequently, 100 μL of N,O-bis-(trimethylsilyl)-trifluoroacetamide containing 1% trimethylchlorosilane was added to the mixture and derivatized at 70 ℃ for 1 h. After that, the solution was cooled in the dark at 25 ℃ for 1 h. Then 600 µL of methyl stearate in heptane (100 ppm) was added. The solution was centrifuged at 13,000 rpm for 10 min to remove precipitates. The supernatant was transferred to a chromatographic vial, and injected into the GC-MS instrument.

### 3.3. GC-MS Analysis

Derivatized samples were analyzed using a GC-MS (QP2020, Shimadzu, Kyoto, Japan). Rtx-5MS with a fused silica capillary column (30 m × 0.25 mm × 0.25 μm, Restek, Bellefonte, PA, USA) was used for the separation of metabolites. The GC-MS temperature was set at 60 ℃ for 1 min, increased to 300 ℃ at a rate of 10 ℃/min, and held for 10 min. GC-MS injector temperature was set at 250 ℃. Its interface and ion source temperatures were 280 ℃ and 230 ℃, respectively. Ionization was achieved with a 70 eV electron beam. The mass spectrometer was programmed under electron impact in a full scan mode from m/z (50–600) with a scan speed of 2 scans/s. Chromatograms and mass spectra were acquired using Shimadzu GC solution (Shimadzu, Kyoto, Japan).

### 3.4. Data Processing

GC-MS data were converted from Shimadzu GC-MS Postrun Analysis to netCDF format file and processed with XCMS web software (https://xcmsonline.scripps.edu). Intensities of features in the data set processed in XCMS were normalized by an internal standard (methyl stearate). PCA and PLS-DA of GC-MS data were performed to visualize the variance of metabolites using SIMCA-P 15.0 (Umetrics, Umea, Sweden). Cross validation was performed using a permutation test that was repeated 200 times. Metabolites with VIP > 1.0 and *p* < 0.05 were considered as metabolites that could discriminate groups. Identification of metabolites was performed by comparing their mass spectra with NIST 14.0. Metabolic pathway analysis (MetPA) was conducted to determine the influence of metabolic pathways on potential marker metabolites using MetaboAnalyst (www.metaboanalyst.ca).

### 3.5. Random Forest Machine Learning Algorithm

A Random Forest machine learning model was generated using metabolites identified in this study. randomForest package in R software (ver. 3.6.0) was used to implement the prediction model, with *mtry* set at 10 and *ntree* set at 500. Plots were made using ggplot2 package.

### 3.6. Statistical Analysis

Differences between groups were examined for their statistical significance using Student’s *t-*test (*p* < 0.05) with SPSS 21.0 (SPSS, Chicago, IL, USA).

## 4. Conclusions

The present study demonstrates that metabolites of GB are clearly dependent on cultivation age and ripening stage. Comprehensive analysis of a wide range of GB metabolites may provide important information about the cultivation age and ripening stage, highlighting that their intrinsic metabolism and metabolic traits might be potentially used as a quality indicator of GB for fruit consumption. This study also shows that a random forest machine learning algorithm could be used as a tool to predict the cultivation age and ripening stage of GB.

## Figures and Tables

**Table 1:** Changes of identified metabolites with different ripening stages in three-year-old GB and four-year-old GB.

Group	Metabolites	Three-Year-Old		Trend ^1)^	Four-Year-Old		Trend
		First Harvest	Fifth Harvest		First Harvest	Fifth Harvest	
Organicacids	Lactic Acid	0.540 ± 0.062	0.370 ± 0.019	↓(***)	0.877 ± 0.052	0.429 ± 0.016	↓(***)
	Glycolic acid	0.150 ± 0.019	0.143 ± 0.008	↓(NS)	0.138 ± 0.009	0.111 ± 0.005	↓(***)
	Malonic acid	1.317 ± 0.146	1.283 ± 0.062	↓(NS)	1.249 ± 0.083	0.825 ± 0.021	↓(***)
	Succinic acid	0.933 ± 0.094	0.626 ± 0.077	↓(***)	0.958 ± 0.049	0.667 ± 0.061	↓(***)
	Glyceric acid	2.160 ± 0.169	2.204 ± 0.112	↑(NS)	1.924 ± 0.085	1.785 ± 0.079	↓(*)
	Malic acid	3.316 ± 0.287	5.310 ± 0.229	↑(***)	3.278 ± 0.116	4.451 ± 0.181	↑(***)
Amino acids	Valine	0.223 ± 0.053	0.237 ± 0.018	↑(NS)	0.319 ± 0.058	0.080 ± 0.022	↓(***)
	Leucine	0.066 ± 0.018	0.095 ± 0.004	↑(NS)	0.080 ± 0.019	0.059 ± 0.004	↓(NS)
	Isoleucine	0.322 ± 0.051	0.284 ± 0.009	↓(NS)	0.242 ± 0.099	0.100 ± 0.052	↓(*)
	Serine	0.252 ± 0.036	0.194 ± 0.011	↓(**)	0.162 ± 0.081	0.103 ± 0.024	↓(NS)
	Threonine	0.195 ± 0.039	0.189 ± 0.015	↓(NS)	0.108 ± 0.044	0.120 ± 0.016	↑(NS)
	Glutamic acid	0.337 ± 0.064	0.239 ± 0.056	↓(*)	0.531 ± 0.046	0.031 ± 0.018	↓(***)
	Tryptophan	0.361 ± 0.093	0.661 ± 0.063	↑(***)	0.581 ± 0.067	0.043 ± 0.020	↓(***)
Sugars & Sugar derivatives	Xylose	0.925 ± 0.285	1.225 ± 0.055	↑(*)	0.587 ± 0.254	0.698 ± 0.218	↑(*)
	Fructose	3.577 ± 0.285	5.635 ± 0.216	↑(***)	2.577 ± 0.163	4.176 ± 0.379	↑(***)
	Glucose	4.859 ± 0.515	9.367 ± 0.501	↑(***)	4.094 ± 0.257	7.163 ± 0.411	↑(***)
	Galactose	1.626 ± 0.234	2.209 ± 0.150	↑(**)	1.054 ± 0.021	2.742 ± 0.151	↑(***)
	Glycerol	1.983 ± 0.158	2.505 ± 0.071	↑(***)	2.411 ± 0.052	2.032 ± 0.090	↓(***)
	Myo-inositol	1.774 ± 0.377	1.290 ± 0.119	↓(NS)	1.111 ± 0.117	2.290 ± 0.158	↑(***)
Fatty acid	Palmitic acid	0.507 ± 0.085	0.505 ± 0.019	↓(NS)	0.521 ± 0.069	0.463 ± 0.008	↓(NS)

1) *p < 0.05; ** *p* < 0.01; *** *p* < 0.001, NS = not significant.

## Supplementary Materials

The following are available online at https://www.mdpi.com/1420-3049/24/21/3837/s1, Table S1. Metabolites data for the prediction model of ripening stage and cultivation age.
